# A Discriminant Distance Based Composite Vector Selection Method for Odor Classification

**DOI:** 10.3390/s140406938

**Published:** 2014-04-17

**Authors:** Sang-Il Choi, Gu-Min Jeong

**Affiliations:** 1. Department of Applied Computer Engineering, Dankook University, 126 Jukjeon-dong, Suji-gu, Yongin-si, Gyeonggi-do 448-701, Korea; E-Mail: choisi@dankook.ac.kr; 2. Electrical Engineering, Kookmin University 2, 861-1, Jeongeung-dong, Songbuk-gu, Seoul 136-702, Korea

**Keywords:** distance discriminant, composite vector, odor classification, sensor array, electronic nose

## Abstract

We present a composite vector selection method for an effective electronic nose system that performs well even in noisy environments. Each composite vector generated from a electronic nose data sample is evaluated by computing the discriminant distance. By quantitatively measuring the amount of discriminative information in each composite vector, composite vectors containing informative variables can be distinguished and the final composite features for odor classification are extracted using the selected composite vectors. Using the only informative composite vectors can be also helpful to extract better composite features instead of using all the generated composite vectors. Experimental results with different volatile organic compound data show that the proposed system has good classification performance even in a noisy environment compared to other methods.

## 1. Introduction

An electronic nose is an instrument intended to identify the specific components of an odor. While human olfactory sensing is prone to be easily fatigued, an electronic nose has the merit of consistently detecting odors, including those harmful to the human body [1–4]. Electronic nose systems are used for various purposes, such as quality control applications in the food and cosmetics industries, the detection of odors regarding specific diseases for medical diagnosis, and the detection of gas leaks for environmental protection [3,5–9].

An electronic nose consists of a sensor array for chemical detection, which is made of polymer carbon composite materials, and a classifier based on various pattern recognition techniques. Hence, the sensitivity of a sensor array and the design of a classifier are crucial factors for the improvement of electronic noses. There are several types of sensor arrays for electronic noses [10–15]. Among them, conducting polymer composites, intrinsically conducting polymer and metal oxides are most commonly used for sensing materials in conductivity sensors. Once volatile organic compounds (VOC) are adsorbed on the sensor surface, a specific response is obtained as a numerical variable by an electronic interface.

In classification problems, the processes can be decomposed into a few steps: feature selection, feature extraction and choosing a classifier. Various static or dynamic information for odor classification can be obtained from the sensor response curve [16–18]. In [17,18], five features, which are the relative change in resistance, the curve integral both over the gas adsorption and desorption process and the phase space integral, again over adsorption and desorption, are extracted from the response curves of six metal oxide sensors. The analysis of the dynamic features of metal oxide sensors was presented to classify four types of volatile compounds, namely acetone, acetic acid, acetaldehyde and butyric acid [16] and active analyses were proposed to deal with gas mixture problems [19,20]. In [21–23], various compensation methods were proposed to solve the drift problem causing a random temporal variation of the sensor response under identical conditions.

The features extracted from the sensor array are fed into a classifier such as the NN (Nearest Neighbor rule) [2] or SVM (Support Vector Machine) [9] for prediction of the class label. In order to improve the performance of a classifier, various feature extraction methods can be used for discriminant analysis and dimensionality reduction [24–27]. Since each method has its pros and cons, an appropriate method must be selected considering the properties of the data and the problem that needs to be solved. For instance, the PCA (Principal Component Analysis) method [28] does not utilize class information of data samples, and finds the projection vectors that correspond to a set of large eigenvalues of the total scatter matrix of data samples. Thus, it is more appropriate to use the PCA method for data representation, rather than data classification. On the other hand, the LDA (Linear Discriminant Analysis) method [29] seeks the linear transformation that maximizes the ratio of the between-class scatter matrix (*S_B_*) and the within-class scatter matrix (*S_w_*). While it gives good performance for classification problem, it suffers from the SSS (Small Sample Size) problem [29] in case of high-dimensional data.

The above methods extract features based on covariance matrices which differ depending on their objective functions. Unlike this, some methods such as MatFLDA (Matrixized Fisher Linear Discriminant Analysis) [30], 2DFLD (Two-Dimensional Fisher Linear Discriminant) [31], or CLDA (Composit LDA) [32,33], use a different type of covariance matrix, which is called an image-covariance matrix. The elements of an image covariance matrix are defined as the expectation of the inner products of predefined vectors. These methods are often effective for data that has a large correlation between primitive variables or high-dimensional data such as the electronic nose data [34] because they utilize information about the statistical dependency among multiple primitive variables and result in a saving in computational effort.

The composite features are extracted by using the covariance of composite vectors composed of a number of primitive variables in various shapes of windows. However, it is likely that there is redundancy between composite vectors when generating composite vectors. Moreover, If there are problems in the data collection process, or when attributes among the collected primitive variables that have no association with solving the classification problem are included, the feature extraction results do not result in optimal solutions and degrade the classification performance [24]. Therefore, distinguishing good composite vectors containing informative primitive variables before the feature extraction process is important to extract better composite features for classification.

**Figure 1.**
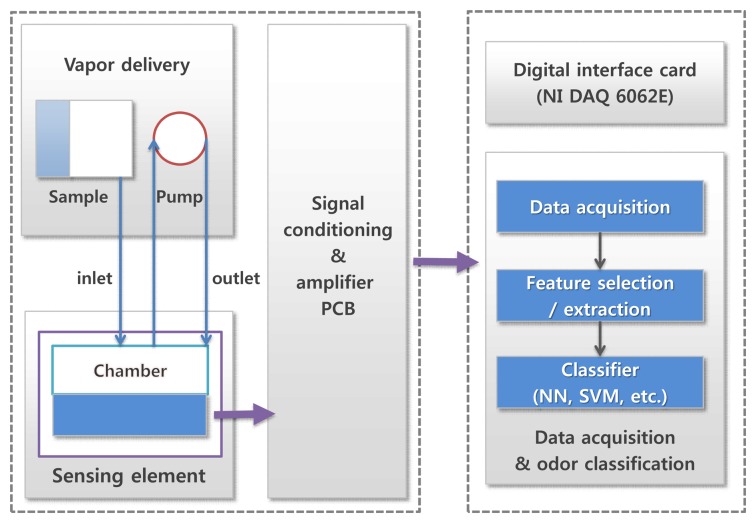
The schematic diagram of our electronic nose system.

In this paper, we propose a method to select the composite vectors which contain informative variables in an electronic nose data sample measured by a sensor array. We measure the amount of discriminative information that each composite vector has, based on the discriminant distance [35] for each composite vector and rank *n_Cf_* composite vectors in descending order according to its discriminant score. The informative composite vectors are distinguished before the process of feature extraction, and then the composite features to be used for the classifier are extracted from the only selected composite vectors. There are potential benefits in employing this selection process such as reduction in computation, storage and processing time in addition to prediction performance improvement. In the process of extracting composite features, the computational effort increases in the order of *υ*^2^ as the number of composite vectors (*υ*) increases. This implies that the computational complexity can be significantly reduced by the proposed method. By using a classifier in an electronic nose with the extracted composite features, we design the robust electronic nose system to noisy environments (Figure 1). The experimental results show that the proposed method gives very good classification results even in a noisy environment.

The rest of this paper is organized as follows. Section 2 introduces a discriminant distance and presents how to select composite vectors based on their discriminant scores. Section 3 explains the acquisition of electronic nose data and how composite features are extracted using the selected composite vectors for odor classification. Section 4 describes the experimental results and the conclusions follow in Section 5.

## 2. Composite Vector Selection Based on Discriminant Distance

**Figure 2.**
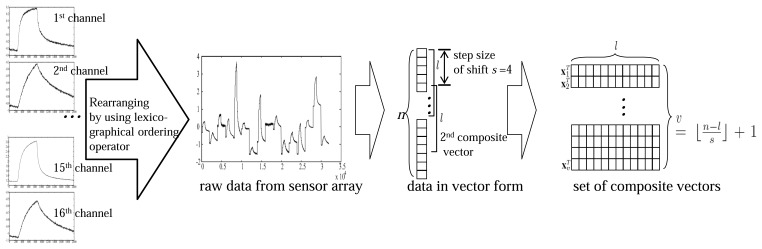
Constructing composite vectors.

Composite vectors can be defined in various ways depending on the shape of a window. The data acquired from a sensor array is stored in an n-dimensional vector, and a composite vector x*_i_* ϵ ℝ *^l^* consists of *l*(*l* < *n*) primitive variables. Composite vectors are generated by shifting a window as much as *s*, which is usually smaller than the length of a composite vector, and thus composite vectors overlap with each other, as shown in Figure 2. The correlation between neighboring variables can be better utilized in the use of the covariance of composite vectors. The number of composite vectors *υ* is $\left\lfloor \frac{n-l}{s}\right\rfloor +1$, where └ · ┘ is the floor operator, which gives the largest integer value that is not greater than the value inside the operator. Then, the *k*-th data sample is represented by *X*(*k*) = [**x**_1_(*k*),..,**x***_υ_*(*k*)]*^T^* ϵ ℝ *^υ^*^×^*^l^*, which is a set of composite vectors. The final composite features for classification are extracted by using the covariance of these composite vectors [36].

However, the overlapped composite vectors as in Figure 2, which may result in redundancy in extracting composite features. Therefore, it needs to find out the composite vectors that promise good class separability among different classes as well as make the samples in the same classes as close as possible. Motivated from the method to select individual variables based on a distance discriminant [35], we define the distance within classes $\left(D_{W}^{i}\right)$ and the distance between classes $\left(D_{B}^{i}\right)$ to compute the discriminant distance for the *i*-th composite vector ${\text{x}}_{i}(k)={\left[x_{i}^{1}(k),x_{i}^{2}(k),..,x_{i}^{l}(k)\right]}^{T}$ as follows:

(1) $$\begin{array}{lll} D_{W}^{i} & = & \overset{l}{\underset{j=1}{\text{∑}}}\overset{c}{\underset{i=1}{\text{∑}}}\frac{1}{\left(N_{i}-1\right)}\underset{x_{i}^{j}(k)\in c_{i}}{\text{∑}}{\left(x_{i}^{j}(k)-m_{i}^{j}\right)}^{2}\\ D_{B}^{i} & = & \overset{l}{\underset{j=1}{\text{∑}}}\overset{c}{\underset{i=1}{\text{∑}}}\frac{N_{i}}{N}{\left(m_{i}^{j}-m^{j}\right)}^{2}. \end{array}$$

Here, $m_{i}^{j}$, *m^j^* and *N_i_* are the *j*-th element of the mean of the class *c_i_*, the *j*-th element of the mean of whole training samples and the number of samples in the class *c_i_*, respectively. Then, the discriminant distance for the *i*-th composite vector is computed by $D_{B}^{i}-\beta D_{W}^{i}$, which reflects the discriminative information of each composite vector. The value of *ß* can be determined depending on the distribution of data samples. For example, in case of the distribution which has good class separability but large variance in the same class, small penalty (*β*) on $D_{W}^{i}$ will be better. By investigating the performance with respect to *β*, we set *β* as 2. For composite vector selection, we define the measure vector as **S** ϵ ℝ *^υ^* whose element $S_{i}=D_{W}^{i}-\beta D_{B}^{i}$. Finally, *n_cf_* composite vectors corresponding to larger *S_i_*s are selected for extracting the final composite features.

## 3. Design of Electronic Nose System

### 3.1. Acquisition of Electronic Nose Data

**Figure 3.**
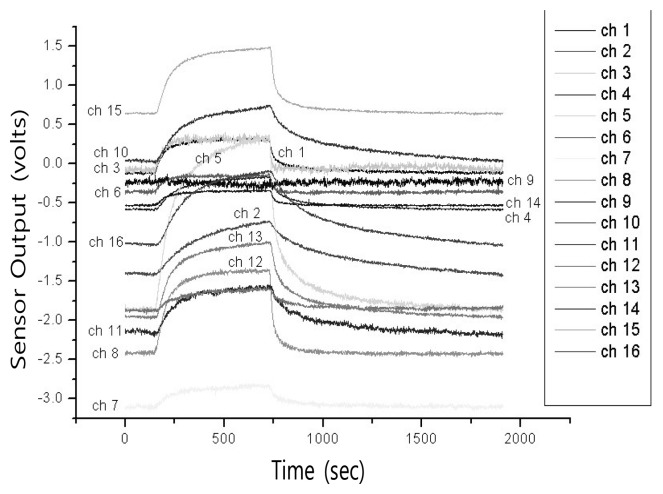
Typical time-responses of 16 channel sensor array with respect to inflow of acetone vapor at 5,000 ppm [2].

The sensor array used in our system was implemented by dispensing a CB polymer composite-solvent solution in a micromachined gas sensor array chip [15]. While the polymer composite has some drawbacks such as sensor drift, limited sensor life, or sensitivity to temperature and humidity it offers many advantages over other materials when used as gas sensor, e.g., the wide range of polymetric materials, inexpensiveness, stable operation at room temperature, and less power consumption, *etc*. [10] The sensor array consists of 16 separate sensors with an interdigitated electrode, microheater, and micromachined membrane in each channel for further temperature-controlled measurement applications (Table 1). The resistance change of each polymer composite film was monitored in response to the incorporation of chemical vapor. The resistance change of polymer composite film was amplified by 20 times and recorded every 0.1 s (Figure 3). Measurement consisted of three steps of stabilization (30 s), exposure (60 s), and purge (110 s). It was performed after the sensor array was placed into the chamber and and the signal of resistance was stabilized. Then, the flow control unit in our system allows the vapors to flow in at desired concentration during about 60 s and afterward flushes the remainder by air flow for about 110 s [37]. The measured data are collected in PC using data acquisition (DAQ) board DAQ6062E and LabVIEW (National Instrumentation, USA). The voltage-divider operated in the range from -10 V to +10 V and gains of 16 identical amplifiers were set to 10 (output/input voltage) for maximum DAQ resolution [15].

### 3.2. Extraction of Composite Features from Selected Composite Vectors

It is very effective for classifying patterns if the within-class variance is small while the between-class variance is large. Similar to LDA, a discriminant analysis using the covariance of composite vectors is derived from the between-class covariance matrix (*C_B_*) and the within-class covariance matrix (*C_W_*) [29]. Assume that each training sample belongs to one of *c* classes, and that there are *N_i_* samples in the class *c_i_*. Let *X′*(*k*) ϵ ℝ *^n_cf_^*^×^*^l^* denote the set of the selected composite vectors of the *k*-th sample. Then, *C_W_* ϵ ℝ *^n_cf_^*^×^ *^n_cf_^* is defined as

(2) $$C_{W}=\sum_{i=1}^{c}p_{i}\{\frac{1}{N_{i}}\sum_{k\in c_{i}}(X^{\prime }(k)-M_{i}){\left(X^{\prime }(k)-M_{i}\right)}^{T}\}$$

where $M_{i}=\frac{1}{N_{i}}\sum_{X^{\prime }(k)\in c_{i}}X^{\prime }(k)$. Here, *p_i_* is a prior probability that a sample belongs to class *c_i_*. *C_B_* ϵ ℝ *^n_cf_^*^×^*^n_cf_^* is also defined as

(3) $$C_{B}=\sum_{i=1}^{c}p_{i}(M_{i}-M){\left(M_{i}-M\right)}^{T}.$$

The image covariance can be also interpreted from another point of view, not from the view of the composite vectors. If letting ***χ***(*k*) and m be column vectors of *X′*(*k*) and *M*, respectively, *C_W_* and *C_B_* can be rewritten as

(4) $$\begin{array}{lll} C_{W} & = & \overset{l}{\underset{j=1}{\text{∑}}}[\overset{c}{\underset{i=1}{\text{∑}}}p_{i}\{\frac{1}{N_{i}}\underset{k\in c_{i}}{\text{∑}}\left(\chi_{j}(k)-{\text{m}}_{j}^{i}){\left(\chi_{j}(k)-{\text{m}}_{j}^{i}\right)}^{T}\}\right]\\ C_{B} & = & \overset{l}{\underset{j=1}{\text{∑}}}[\overset{c}{\underset{i=1}{\text{∑}}}p_{i}({\text{m}}_{j}^{i}-{\text{m}}_{j}){\left({\text{m}}_{j}^{i}-{\text{m}}_{j}\right)}^{T}]. \end{array}$$

*χ_j_*(*k*) consists of the j-th elements in each of the selected composite vectors, which is sampled from *X′*(*k*) with regularly varying intervals. This is the similar effect that generates *l* times more data samples of smaller size. The increase of the number of data samples will provide a robust performance to the variation caused by the noise.

Composite features are obtained by linear combinations of the composite vectors and each feature is a vector whose dimension is equal to the dimension of the composite vector. For composite feature extraction, the projection matrix *W* is found by maximizing the following objective function:

(5) $$W=\arg\underset{W}{\max}\frac{\left|W^{T}C_{B}W\right|}{\left|W^{T}C_{W}W\right|}.$$

The set of composite features for *Y*(*k*) is obtained by projecting *X′*(*k*) into the projection matrix *W* as

(6) $$\begin{array}{cc} Y(k)=W^{T}X^{\prime }(k), & k=1,2,\ldots ,N, \end{array}$$

where *Y*(*k*) ϵ ℝ *^m×l^* has *m* composite features [**y**_1_(*k*) … **y***_m_*(*k*)]*^T^*.

The length of the window (*l*), the number of composite features (*m*) and the step size of the shift (*s*) are important parameters that influence the classification performance. We investigated the classification rates with respect to *l*, *m* and *s*. Table 2 shows the classification rates with respect to *l* and *m*. In this case, we set *s* = *l*/2 as in [32]. As can be seen in Table 2, the classification rates are not sensitive to *l* if *m* is properly decided. We set *l* and *m* to 400 and 25, respectively. Then, we investigated the classification rates with respect to *s*. As can be seen in Table 3, the classification rates are not sensitive to *s* and the classification rate of *s* = 200 was slightly better than those of other *s* values. Therefore, we set *s* to 200. Also, in order to find the optimal number of the selected composite vectors, we checked the classification rates for the electronic nose data by increasing the number of selected composite vectors *n_cf_*. As a result, we set the number of selected composite vector *n_cf_* to 150.

**Figure 4.**
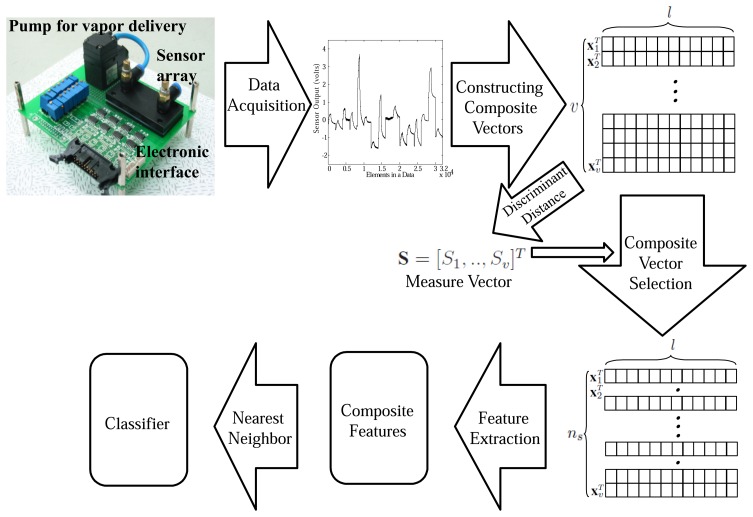
Overall procedure of the proposed electronic nose system.

The overall procedure of our system can be summarized as follows (Figure 4):

(1) Generate *υ* composite vectors **x***_i_*(*k*), *i* = 1,.., *υ* ϵ ℝ *^l^* from an e-nose data sample by shifting the *l* length of window as much as the step size of shift (*s*).
(2) For each composite vector **x***_i_*(*k*), compute the distances within- $\left(D_{W}^{i}\right)$ and between-classes $\left(D_{B}^{i}\right)$.
(3) Compute the discriminant distance for the *i*-th composite vector by $S_{i}=D_{B}^{i}-\beta D_{W}^{i}$.
(4) Construct the measure vector **S** ϵ ℝ *^υ^* whose element *S_i_*.
(5) Select *n_cf_* composite vectors corresponding to larger *S_i_*s.
(6) Extract the final composite features with the only selected composite vectors.

## 4. Experimental Results

**Figure 5.**
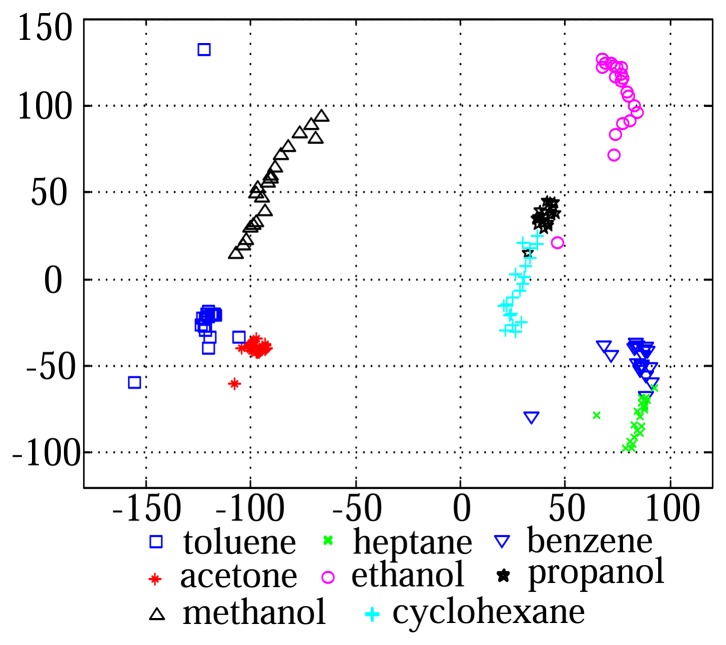
Distribution of the data samples in the Principal Component Analysis (PCA) feature space.

The VOC measurement data consists of 8 classes, which are acetone, benzene, cyclo-hexane, ethanol, heptane, methanol, propanol, and toluene [15]. For each class, we obtained 20 samples, and thus the total data set contains 160 samples. Figure 5 shows the distribution of the data samples in the subspace consisted of two principal component axes. The e-nose sensor used in this experiment measures vapors with a speed of 10 Hz, which corresponds to a sampling rate of 2,000 points per 200 s. Each data sample was measured through 16 channel over 2,000 time points and was represented as a 16 × 2,000 matrix. Then, the raw data was transformed into the 32,000-dimensional vector by using the lexicographic ordering operator for feature extraction (Figure 2).

**Figure 6.**
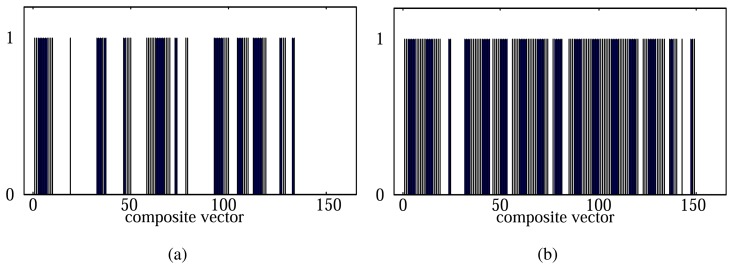
Distribution of the selected composite vectors. (**a**) 60 composite vectors. (**b**) 120 composite vectors.

When setting *l* and *s* as 400 and 200, respectively, the total 159 composite vectors can be generated from a 32,000-dimensional data sample. We measured the discriminant scores of each composite vector by using the proposed method. Out of the total 159 composite vectors, we represented the composite vectors with top 60 and 120 scores as ‘1’ and the rest as ‘0’ (Figure 6). In Figure 6, we can see that the ‘stabilization’ and ‘purge’ periods contain the discriminative information for odor classification as well together with the ‘exposure’ period.

We compared the classification performance of the proposed method (CVS) with that of the LDA method [26], the FF (Feature Feedback) method [38], the CC-PCA (Component Correction by PCA) method [39], and CC-CPCA (Component Correction by Common PCA) method [22]. We applied PCA after CC-PCA and CC-CPCA, which slightly increased their classification rates. Each method was evaluated using an 8-fold cross validation strategy [40]. In this scheme, the data is first randomly partitioned into 8 equally sized folds. Then, 8 iterations of training and testing are performed, within each of which a different fold of the data (20 data samples) is used for testing, while the remaining 7 folds (140 data samples) are used for training. The nearest neighbor rule was used as a classifier and the *l_2_* nor was used to measure the distance between two samples. We repeated this test 8 times and computed the average classification rate. All the data samples are normalized using the mean and the variance of the training set.

**Figure 7.**
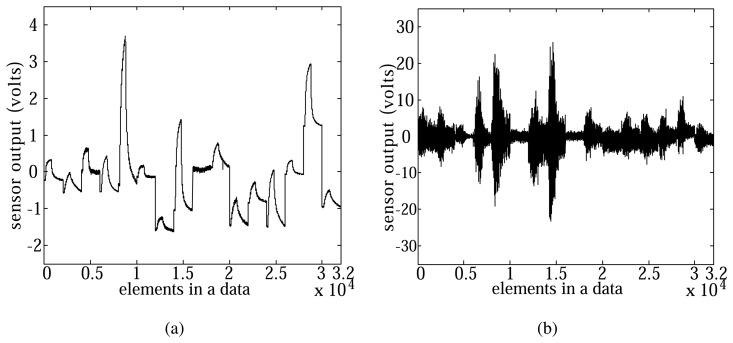
Electronic nose data w/o and with Gaussian noise. (**a**) Electronic nose data without noise. (**b**) Data with Gaussian noise (std 3).

**Figure 8.**
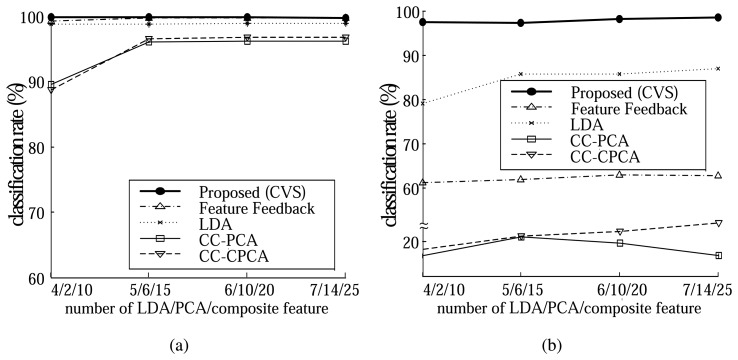
Classification rates for the electronic nose data. (**a**) Classification rates for the original electronic nose data. (**b**) Classification rates for the electronic nose data with Gaussian noise (std 3).

Since noise is likely to occur in sensing data, we added Gaussian noise with a standard deviation 3 to each data sample, and evaluated the robustness of each method to the noise (Figure 7). Figures 8 show examples of the data with or without Gaussian noise and the classification rates of each case, respectively.

For the original data, all the methods classified each vapor well with high classification rates as can be seen in Figure 8a. When Gaussian noise is added, the classification rates of the other methods decreased rapidly (Figure 8b). In contrast, the proposed method gave consistently high classification rates of 97.3% ∼ 98.4%, which showed that our system performs reliably in a noisy environment.

## 5. Conclusions

We have presented a method to select useful composite vectors for odor classification. Composite vectors, which are generated from an electronic nose data sample by shifting the window, are likely to contain redundant information for extracting discriminant features and some noise occurred in measuring with a sensor array. Thus, we evaluated the class separability power of each composite vector based on a discriminant distance and selected the only composite vectors with large discriminative information. This selection process has the advantage to holistically view the electronic nose response by its focus on the extraction of informative response characteristics. The proposed composite vector selection method not only reduced the computational complexity, but also helped to extract better features. Since extracting good features not only relieves the influence of noise in the measured data, but also improves the performance of a classifier such as SVM and NN. When using SVM without any feature extraction, while the classification rate for the original electronic nose data was 98.0%, the classification rate dropped to 51.2% for the data with Gaussian noise. On the contrary, NN with the features extracted by the proposed method gave the classification rates of 99.8% and 98.4% for the same data sets, respectively. Hence, the proposed method can be utilized together with algorithms of other classification processes such as feature selection or classifier design and improve the performance of the overall classification system.

In this paper, we focus on the classification between gas data classes without interference. It is also important to classify the data which contains combinations of gases, different concentration, *etc.* in e-nose data. In near future, we will deal with the interference between gases and gas combinations.

## Figures and Tables

**Table 1.** The list of 16 CB polymer composites used in the sensor array.

Number	Polymer I.D.
Ch1	Poly(methyl methacrylate)
Ch2	Polyvinylpyrrolidone
Ch3	Poly(vinyl acetate)
Ch4	Poly(ethylene oxide)
Ch5	Polycaprolactone
Ch6	Poly(4-methylstyrene)
Ch7	Poly(styrene-co-methyl methacrylate)
Ch8	Poly(ethylene-co-vinylacetate)
Ch9	Poly(bisphenol A carbonate)
Chl0	Poly(4-vinyl pyridine)
Chll	Poly(vinyl butyral)-co-vinyl alcphol-co-vinyl acetate
Chl2	Poly(vinyl stearate)
Chl3	Ethyl cellulose
Chl4	Polystyrene-black-polyisoprene-black-polystyrene
Chl5	Hydroxypropyl cellulose
Chl6	Cellulose acetate

**Table 2.** Classification rates with respect to *l* and *m*.

*m*	1	3	5	11	16	21	26	31	36
*l*									
100	67.5	91.9	98.1	98.1	98.1	98.1	98.1	98.1	98.1
200	72.5	91.9	98.1	98.1	98.1	98.1	98.1	98.1	98.1
400	78.8	94.4	98.8	98.1	98.8	98.1	98.8	98.8	98.8
800	71.3	95.6	98.8	98.8	98.1	98.1	98.1	98.1	98.1
1600	64.4	75.0	98.8	97.5	98.1	98.1	98.1	98.1	98.1

**Table 3.** Classification rates with respect to *s*.

*s*	50	75	100	125	150	175	200	225	250
Classi. rate	98.1	98.1	98.1	98.1	98.1	98.1	98.1	98.8	98.1
